# Supplementary material for: NSUN2 mediates intestinal stem cell expansion and colorectal tumour initiation via MAPK/ERK signalling
Source: Cell Death Dis. 2026 Mar 19;17(1):322. doi: 10.1038/s41419-026-08560-0 (PMC13039175; doi:10.1038/s41419-026-08560-0)
Supplement: Supplementary file 1 — Supplementary legends and materials [file 41419_2026_8560_MOESM1_ESM.pdf]

Supplementary Fig S1. RNA metabolic processes are enriched following *Apc*-loss and NSUN2 is upregulated in *Apc*-deficient mouse intestinal tissues

A Validation of *Apc*-loss via nuclear β-CATENIN staining in wild-type and $Apc^{fl/fl}$ mouse small intestines by immunohistochemistry.

B Validation of Apc-loss via phosphorylated β-CATENIN protein levels in wild-type and $Apc^{fl/fl}$ mouse colon tissue by western blot.

C mRNA expression levels (FPKM values) of genes involved in m^5C methylation (writers, readers and erasers) in RNA-seq from wild-type and $Apc^{fl/fl}$ mouse small intestines collected post day 5 Tamoxifen induction (data are presented as mean ± SD; ***p: 0.0007, p: 0.0017; two-tailed t-test, n= 3 vs 3 biologically independent mice).

D Quantification of normalised protein expression levels in wild-type and $Apc^{fl/fl}$ mouse small intestine, using β-ACTIN loading control (data are presented as mean ± SD; **p: 0.0044; two-tailed t-test, n= 3 vs 3 biologically independent mice).

E Quantification of normalised protein expression levels in wild-type and $Apc^{fl/fl}$ mouse colon, using β-ACTIN loading control (data are presented as mean ± SD; *p: 0.0279; two-tailed t-test, n= 3 vs 3 biologically independent mice).

F *Nsun2* expression in stem cell and non-stem cell intestinal population based on a stem cell marker, EphB2. EphB2 low, medium, and high cell populations were sorted by FACs based on EphB2 expressions in wild-type mouse small intestine (data are presented as mean ± SD; One-way ANOVA test, n= 5 vs 3 vs 5 biologically independent mice).

Supplementary Fig S2. NSUN2 is overexpressed in patients with truncated *APC* mutation. But NSUN2 expression is not changed in *KRAS*/*P53* mutant patients, or cancer stages and TNM.

A *NSUN2* mRNA expression in primary tumours with mutations in *APC (*truncated*) (left)*, *KRAS (middle)*, and *P53 (right)* genes from TCGA COAD-READ PanCancer patients compared to APC^{wt} primary tumours in cBioportal. (data are presented as mean ± SD; **p: 0.0017, *p: 0.0279; two-tailed t-test, APC^{wt} vs $APC^{Truncated}$ n= 141 vs 385 patients, $KRAS^{wt}$ vs $KRAS^{mut}$ n= 310 vs 212, $P53^{wt}$ vs $P53^{mut}$ n= 213 vs 309).

B *NSUN2* mRNA expression in patients with different cancer stages from TCGA COAD-READ PanCancer dataset in cBioportal.

C *NSUN2* mRNA expression in patients with different TNM categories from TCGA COAD-READ PanCancer dataset in cBioportal.

Supplementary Fig S3. *Nsun2* knockdown in $Apc^{fl/fl}$ mouse small intestinal organoids validated by qPCR

A Validation of *Nsun2* knockdown in $Apc^{fl/fl}$ mouse small intestinal organoids by qPCR (left) (data are presented as mean ± SD; ****p< 0.0001) and by western blot (middle, right) (data are presented as mean ± SD; ****p< 0.0001).

Supplementary Fig S4. Reduction in other stem cell marker genes after *Nsun2*-deletion and validation of NSUN2 deletion in intestinal crypt culture.

A mRNA expression levels of other stem cell marker genes, *Ascl2, Ephb2, Igfbp4, Lrig1,* and *Smoc2*. All mRNA expressions were normalised to *β-actin*, a housekeeping control. (data are presented as mean ± SD; two-tailed t-test, ***p< 0.0001, **p< 0.001 , *p< 0.05, not significant: ns, (n= 4 vs 4 vs 3 vs 4 biologically independent mice).

B Quantification of number of Lgr5 spots per crypt by RNAscope in Vil-CreERT2 wild-type, *Nsun2*$^{fl/fl}$, *Apc*$^{fl/fl}$ and, *Apc*$^{fl/fl}$ *Nsun2*$^{fl/fl}$ mouse small intestines.

C Quantification of Lgr5 spots mean intensity per crypt by RNAscope in Vil-CreERT2 wild-type, *Nsun2*$^{fl/fl}$, *Apc*$^{fl/fl}$ and, *Apc*$^{fl/fl}$ *Nsun2*$^{fl/fl}$ mouse small intestines.

D Images of western blotting analysis for NSUN2 and a house-keeping control VINCULIN protein expressions in *Apc*$^{fl/fl}$ and *Apc*$^{fl/fl}$ *Nsun2*$^{fl/fl}$ Vil-CreERT2 mouse small intestinal crypt cultures.

E-F Representative images of clonogenicity assay (left) performed in direct crypt culture from *Apc*$^{fl/fl}$, *Apc*$^{fl/fl}$ *Nsun2*$^{fl/fl}$ Vil-CreERT2 mouse small intestines. 50,000 single cells seeded on Day 1 and relative cell viability (%) (right) was measured by 24h Resazurin incubation on Day 6 (data are presented as mean ± SD; *p: 0.0438; Unpaired t-test with Welch's correction, n = minimum 3 biologically independent mice per group).

Supplementary Fig S5. *Nsun2*-loss do not affect transcriptome of normal intestine.

A Heat-map of differentially expressed intestinal stem cell markers and Wnt targets comparing small intestinal crypts from *Vil-Wt* vs *Vil-Nsun2* mice (n= 3 vs 3 biologically independent mice).

B Schematic demonstration of *Apc* and *Nsun2* knockout mouse model generation in the intestinal tissue-specific *Lgr5-IRES-eGFP-CreERT2* line. The figure was created by Biorender.com.

Supplementary Fig S6. mRNA methylation profile in all experimental groups and distribution and sequence context of 525 common m^{5}C sites between *in vivo* and *in vitro ApcNsun2* deficient models

A Heat-map of methylation profile in all experimental models used in whole transcriptome bisulphite sequencing. 13,153 m^{5}C sites for *Vil-Wt* vs *Vil-Apc* mice, 12,754 m^{5}C sites for *Vil-Wt* vs *Vil-*

Nsun2 mice, 13,374 m^{5}C sites for *Vil-Apc* vs *Vil-ApcNsun2* mice are identified (n= 3 vs 3 biologically independent mice). 11,134 m^{5}C sites for *Apc$^{fl/fl}$-shCtrl* and *Apc$^{fl/fl}$-shNsun2* are identified (n= 3 experimental replicates). Differentially methylated sites (DMSs) were defined using Δmethylation ration: ± 0.05.

B The bar graph represents distribution of 525 common m^{5}C sites detected as a result of overlapping vivo and vitro m^{5}C sites in different mRNA regions.

C The probability pattern represents sequence context of 10 bases up and downstream of hypo-methylated (295) and hyper-methylated (235) of common 525 NSUN2-dependent m^{5}C sites based on located in different mRNA regions.

Supplementary Fig S7. NSUN2 knockdown reduces clonogenic capacity of *Apc$^{fl/fl}$; P53$^{fl/fl}$* but not *Apc$^{fl/fl}$; Kras$^{G12D/+}$* mouse colorectal cancer organoids

A Quantification of normalised NSUN2 protein expression levels in *Apc$^{fl/fl}$; P53$^{fl/fl}$ (AP)-shCtrl* and *Apc$^{fl/fl}$; P53$^{fl/fl}$ (AP)-shNsun2* mouse small intestinal organoids by western blot, using β-ACTIN loading control (data are presented as mean ± SD; ****p< 0.0001 (*shCtrl* and *shNsun2-1*), ****p< 0.0001 (*shCtrl* and *shNsun2-2*); One-way ANOVA test comparing to *shCtrl*, n= 3 experimental replicates).

B The percentage of organoid number, diameter, and area in *shNsun2-1* and *shNsun2-2* groups compared to *shCtrl* in *Apc$^{fl/fl}$; P53$^{fl/fl}$ (AP)* mouse small intestinal organoids (data are presented as mean ± SD; ***p< 0.0001, **p< 0.001 , *p< 0.05, Ordinary One-way ANOVA test compared to *shCtrl,* n= 6 experimental replicates).

C Quantification of normalised NSUN2 protein expression levels in *Apc$^{fl/fl}$; Kras$^{G12D/+}$ (AK)-shCtrl* and *Apc$^{fl/fl}$; Kras$^{G12D/+}$ (AK)-shNsun2* mouse small intestinal organoids by western blot, using β-ACTIN loading control (data are presented as mean ± SD; ***p: 0.0001 (*shCtrl* and *shNsun2-1*), ***p: 0.0002 (*shCtrl* and *shNsun2-2*); One-way ANOVA test comparing to *shCtrl*, n= 3 experimental replicates).

D The percentage of organoid number, diameter, and area in *shNsun2-1* and *shNsun2-2* groups compared to *shCtrl* in *Apc*$^{fl/fl}$*; Kras*$^{G12D/+}$ *(AK)* mouse small intestinal organoids (data are presented as mean ± SD; ***p< 0.0001, **p< 0.001 , *p< 0.05, not significant values are not indicated, Ordinary One-way ANOVA test compared to *shCtrl,* n= 5 experimental replicates).

Supplementary Fig S8. MAPK activation rescues clonogenic activity following NSUN2 depletion across multiple, independent models; AKP and KPN metastatic mouse colorectal cancer organoids.

A Representative images of *Apc*$^{fl/fl}$*; Kras*$^{G12D/+}$*; P53*$^{fl/fl}$ *(AKP)* metastatic mouse colorectal cancer organoids for Puromycin selection and clonogenicity (1000 single cells) following *Nsun2* knockdown.

B Validation of *Nsun2* knockdown in *Apc*$^{fl/fl}$*; Kras*$^{G12D/+}$*; P53*$^{fl/fl}$ *(AKP)* metastatic mouse colorectal cancer organoids by qRT-PCR (data are presented as mean ± SD; Ordinary One-way ANOVA test compared to *shCtrl*, n= 4 experimental replicates).

C The percentage of raw clonogenicity levels in *shNsun2-1* and *shNsun2-2* groups compared to *shCtrl* in *AKP* mouse organoids. The percentage of relative viability by Resazurin assay in *shNsun2-1* and *shNsun2-2* compared to *shCtrl* in *AKP* mouse organoids (data are presented as mean ± SD; Ordinary One-way ANOVA test compared to *shCtrl,* n= 3 experimental replicates).

D mRNA expression levels of stem cell markers *(Lgr5, Ascl2, Igfbp4, Lrig1, Smoc2)* regarding ISC signature, respectively, in AKP mouse organoids following *Nsun2*-knockdown. All mRNA expressions were normalised to *β-actin*, a housekeeping control and *shCtrl*. All data are presented using Ordinary one-way ANOVA compared to *shCtrl* statistical test, n≥ 3 experimental replicates.

E Representative images of *Kras*$^{G12D/+}$*; P53*$^{fl/fl}$*; Notch*^{ICD} *(KPN)* metastatic mouse colorectal cancer organoids for Puromycin selection and clonogenicity (1000 single cells) following *Nsun2* knockdown.

F Validation of *Nsun2* knockdown in *Kras*$^{G12D/+}$*; P53*$^{fl/fl}$*; Notch*^{ICD} *(KPN)* metastatic mouse colorectal cancer organoids by qRT-PCR (data are presented as mean ± SD; Ordinary One-way ANOVA test compared to *shCtrl*, n = 4 experimental replicates).

G The percentage of raw clonogenicity levels in *shNsun2-1* and *shNsun2-2* groups compared to *shCtrl* in *KPN* mouse organoids. The percentage of relative viability by Resazurin assay in *shNsun2-1* and *shNsun2-2* compared to *shCtrl* in *KPN* mouse organoids (data are presented as mean ± SD; Ordinary One-way ANOVA test compared to *shCtrl,* n = 3 experimental replicates).

H mRNA expression levels of stem cell markers *(Lgr5, Ascl2, Igfbp4, Lrig1, Smoc2)* regarding ISC signature, respectively, in KPN mouse organoids following *Nsun2*-knockdown. All mRNA expressions were normalised to *β-actin*, a housekeeping control and *shCtrl*. All data are presented using Ordinary one-way ANOVA compared to *shCtrl* statistical test, n≥ 3 experimental replicates.

Supplementary Fig S9. *Nsun2* depletion in *Braf* driven advanced colorectal cancer mouse organoids leads to a modest reduction in clonogenic capacity.

A Representative images of *Braf*$^{V600E/+}$*; P53*$^{fl/fl}$*; Notch*^{ICD} *(BPN)* metastatic mouse colorectal cancer organoids for Puromycin selection and clonogenicity (1000 single cells) following *Nsun2* knockdown.

B mRNA expression levels of *Nsun2* and a stem cell marker *(Lgr5)* in *BPN* mouse organoids following *Nsun2*-knockdown. All mRNA expressions were normalised to *β-actin*, a housekeeping control and *shCtrl*. All data are presented using Ordinary one-way ANOVA compared to *shCtrl* statistical test, n= 4 experimental replicates.

C The percentage of raw clonogenicity levels in *shNsun2-1* and *shNsun2-2* groups compared to *shCtrl* in *BPN* mouse organoids. The percentage of relative viability by Resazurin assay in *shNsun2-1* and *shNsun2-2* compared to *shCtrl* in *BPN* mouse organoids (data are presented as mean ± SD; Ordinary One-way ANOVA test compared to *shCtrl,* n= 4 experimental replicates).

Supplementary Table S1. RNA sequencing data of *Apc*$^{fl/fl}$ and *Apc*$^{fl/fl}$ *Nsun2*$^{fl/fl}$ mouse small intestines.

Supplementary Table S2. RNA sequencing data of *wild-type* and *Nsun2*$^{fl/fl}$ mouse small intestines.

Supplementary Table S3. mRNA bisulphite sequencing m5C calling table in *in vivo* (in Vil-CreERT2 wild-type, *Nsun2*$^{fl/fl}$, *Apc*$^{fl/fl}$ and, *Apc*$^{fl/fl}$ *Nsun2*$^{fl/fl}$ mouse small intestines) and *in vitro* (*Apc*$^{fl/fl}$*-shCtrl* and *Apc*$^{fl/fl}$*-shNsun2* organoid) samples.

Supplementary Table S4. mRNA bisulphite sequencing bisulphite conversion table in *in vivo* (in Vil-CreERT2 wild-type, *Nsun2*$^{fl/fl}$, *Apc*$^{fl/fl}$ and, *Apc*$^{fl/fl}$ *Nsun2*$^{fl/fl}$ mouse small intestines) and *in vitro* (*Apc*$^{fl/fl}$*-shCtrl* and *Apc*$^{fl/fl}$*-shNsun2* organoid) samples for luciferase mRNA spike-in control and ribosomal DNA.

Supplementary Table S5. mRNA bisulphite sequencing differentially methylated sites table in Vil-CreERT2 wild-type and *Nsun2*$^{fl/fl}$ mouse small intestines.

Supplementary Table S6. mRNA bisulphite sequencing differentially methylated sites table in Vil-CreERT2 wild-type and *Apc*$^{fl/fl}$ mouse small intestines.

Supplementary Table S7. mRNA bisulphite sequencing differentially methylated sites table in Vil-CreERT2 *Apc*$^{fl/fl}$ and *Apc*$^{fl/fl}$ *Nsun2*$^{fl/fl}$ mouse small intestines.

Supplementary Table S8. mRNA bisulphite sequencing differentially methylated sites table in *Apc*$^{fl/fl}$*-shCtrl* and *Apc*$^{fl/fl}$*-shNsun2* mouse small intestine organoids.

Supplementary Table S9. 525 common differentially methylated m5C sites between in vivo and in vitro experimental groups in mRNA bisulphite sequencing.

Supplementary Table S10. Gene expression profile of selected differentially hypomethylated genes in RNA sequencing data from Vil-CreERT2 *Apc*$^{fl/fl}$ and *Apc*$^{fl/fl}$ *Nsun2*$^{fl/fl}$ mouse small intestines.

Supplementary Table S11. List of antibodies used in this study.

<table>
<tr><th colspan="2">Protein</th><th>Company</th><th>Catalogue number</th><th>Solution</th><th>Primary antibody dilution</th></tr>
<tr><td rowspan="7">Western blot</td><td>NSUN2</td><td>ThermoFisher</td><td>#PA5-58155</td><td>5% milk</td><td>1:2000</td></tr>
<tr><td>β-ACTIN</td><td>Cell signalling</td><td>#4970S</td><td>5% milk</td><td>1:5000</td></tr>
<tr><td>c-MYC</td><td>Cell signalling</td><td>#5605</td><td>5% milk</td><td>1:200</td></tr>
<tr><td>Phospho-ERK1/2</td><td>Cell signalling</td><td>#4370S</td><td>5% milk</td><td>1:1000</td></tr>
<tr><td>Total-ERK1/2</td><td>Cell signalling</td><td>#4695</td><td>5% milk</td><td>1:1000</td></tr>
<tr><td>Active β-CATENIN</td><td>Cell signalling</td><td>#8814S</td><td>5% milk</td><td>1:1000</td></tr>
<tr><td>Phosphorylated β-CATENIN</td><td>Cell signalling</td><td>#9565</td><td>5% milk</td><td>1:1000</td></tr>
<tr><td>Dot blot</td><td>m^5C</td><td>Active Motif</td><td>#39649</td><td>LICOR+ 0.1% Tween20</td><td>1:3000</td></tr>
<tr><td rowspan="9">IHC</td><td rowspan="3">NSUN2</td><td rowspan="3">ThermoFisher</td><td rowspan="3">#PA5-58155</td><td>1X Citrate buffer pH:6</td><td rowspan="3">1:5000</td></tr>
<tr><td>5% Goat serum</td></tr>
<tr><td>VECTASTAIN®Elite ABC-HRP Kit, Peroxidase (Rabbit IgG)</td></tr>
<tr><td rowspan="3">BrdU</td><td rowspan="3">BD Biosciences</td><td rowspan="3">#347580</td><td>1X Citrate buffer pH:6</td><td rowspan="3">1:500</td></tr>
<tr><td>5% Goat serum</td></tr>
<tr><td>Dako Kit EnVision+System-HRP Anti-Mouse</td></tr>
<tr><td rowspan="3">β-CATENIN</td><td rowspan="3">BD Biosciences</td><td rowspan="3">#610154</td><td>Tris EDTA</td><td rowspan="3">1:50</td></tr>
<tr><td>1% BSA</td></tr>
<tr><td>Dako Kit EnVision+System-HRP Anti-Mouse</td></tr>
</table>

Supplementary Table S12. List of materials used in this study.

Product	Company	#Catalogue number
**Countess Cell Counting Chamber Slides**	Life Technologies	#C10228
**Puromycin**	Gibco	#A11138-03
**Valproic Acid**	Sigma	#PHR1061-1G
**StemProAccutase**	Gibco	#A11105-01
**BSA**	Sigma	#A2153-500G
**Y27632**	Tocris	#1254/10
**BME-2 phenol red**	Biotechne	#3533-010-02P
**Hexadimethrine bromide (Polybrene)**	Sigma	#H9268-5G
**TrypIE Express Phenol Red**	Life Technologies	#12605010
**Resazurin**	Bio-techne	#AR002
**RNeasy Mini Kit**	Qiagen	#74106
**RnaseZap**	Invitrogen	#AM9780
**RNase-Free DNase Set**	Qiagen	#79254
**Dynabeads™ mRNA Purification Kit**	Life Technologies	#61006
**qScript cDNA SuperMix**	vwr	#95048-100
**Sybr green**	Life Technologies	#4472920
**RIPA Buffer**	Sigma	#R0278
**Phosphatase Inhibitor Cocktail**	Sigma	#P0044
**Protease Inhibitor Cocktail**	Sigma	#P8340
**Precision Plus Protein™ Dual Color Standards**	Biorad	#1610374
**Gel Loading Buffer II (Denaturing PAGE)**	Life Technologies	#AM8546G
**NuPage sample reducing agent 10x (DTT)**	Life Technologies	#NP0004
**Nupage 4-12% 1.0 mm 10 wells**	Life Technologies	#NP0321BOX
**Nupage 4-12% 1.5mm 15 wells**	Life Technologies	#NP0336BOX
**NuPage 3-8% Tris-Acetate protein gel**	Life Technologies	#EA0375BOX
**Tris-Acetate SDS buffer**	Life Technologies	#LA0041
**NuPage Mops Running Buffer**	Invitrogen	#NP0001
**Amersham Protran NC 0.45um Nitrocellulose**	Amersham	#15259794
**Nupage Transfer Buffer**	Invitrogen	#NP00061
**Ponceau S solution BioReagent**	Sigma	#P7170-1L
**Thermo Scientific PiERCe ECL Plus Western Blotting Substrate, Conjugate: HRP**	Thermo	#32132
**SuperSignal® West FEMTO Max. Sensitivity Substrate**	Thermo	#PIER34095

**ECL Western blotting substrate**	vwr	#PIER32106
**ReBlot Plus Strong Antibody Stripping Solution (10X)**	Merck	#2504
**Skimmed milk powder**	Marvel	-
**LICOR IntERCept® (PBS) Blocking Buffer**	Li-cor biosciences	#927-70001
**Amersham Hybond-N+ nylon membrane**	Sigma	#RPN203B
**Xylene**	Fisher chemicals	#X/0250/17
**4% formaldehyde**	Fisher Scientific Ltd	#16330638
**H_2O_2**	Scientific lab	#CHE2200
**Citrate Buffer pH:6 10X**	Sigma	#C9999-100mL
**Goat serum**	sigma	#g9023
**Immedge pen**	Vectorlabs	H-4000
**DAB Quanto**	Thermo	#12693967
**DPX**	Cell path	#SEA-1304-00A
**Luciferase control RNA**	Promega Corporation	#L4561
**EZ RNA methylation Kit**	Zymo	#R5001
**10mM HEPES**	Gibco	#15630-056
**B27**	Invitrogen	#12587-010
**N2**	Invitrogen	#17502-048
**EGF**	Peprotech	#AF-100-15
**Noggin**	Peprotech	#250-38
**Nsun2 mouse shRNA**	Horizon discovery	RMM3981-201810604, RMM3981-201837916
**Advanced DMEM/F12 (ADF) media**	Invitrogen	#12634-028

Supplementary Table S13. List of primers and sequences used in this study.

Gene	Company	5' → 3'
Nsun2 (exon6)	IDT	F: ATGTGTGCAGCCCCTGGA
		R: TGGAAAGGGCACACTCATGT
β-Actin	IDT	F: GGCTGTATTCCCCTCCATCG
		R: CCAGTTGGTAACAATGCCATGT
Lgr5	IDT	F: CCTACTCGAAGACTTACCCAGT
		R: GCATTGGGGTGAATGATAGCA
Ascl2	IDT	F: AAGCACACCTTGACTGGTACG
		R: AAGTGGACGTTTGCACCTTCA

Ephhb2	IDT	F: GCCGTGGAAGAAACCCTGAT
		R: GTTCATGTTCTCGTCGTAGCC
Igfbp4	IDT	F: AGAAGCCCCTGCGTACATTG
		R: TGTCCCCACGATCTTCATCTT
Lrig1	IDT	F: TTGAGGACTTGACGAATCTGC
		R: CTTGTTGTGCAAAAAGAGAG
Smoc2	IDT	F: AGTGGAGACATTGGCAAGAAG
		R: ACACACTTTTTGGGCTTGGATT
18S rRNA	IDT	F: CTCAACACGGGAAACCTCAC
		R: CGCTCCACCAACTAAGAACG
