# Supplementary figures and images for: NSUN2 mediates intestinal stem cell expansion and colorectal tumour initiation via MAPK/ERK signalling
Source: Cell Death Dis. 2026 Mar 19;17(1):322. doi: 10.1038/s41419-026-08560-0 (PMC13039175; doi:10.1038/s41419-026-08560-0)

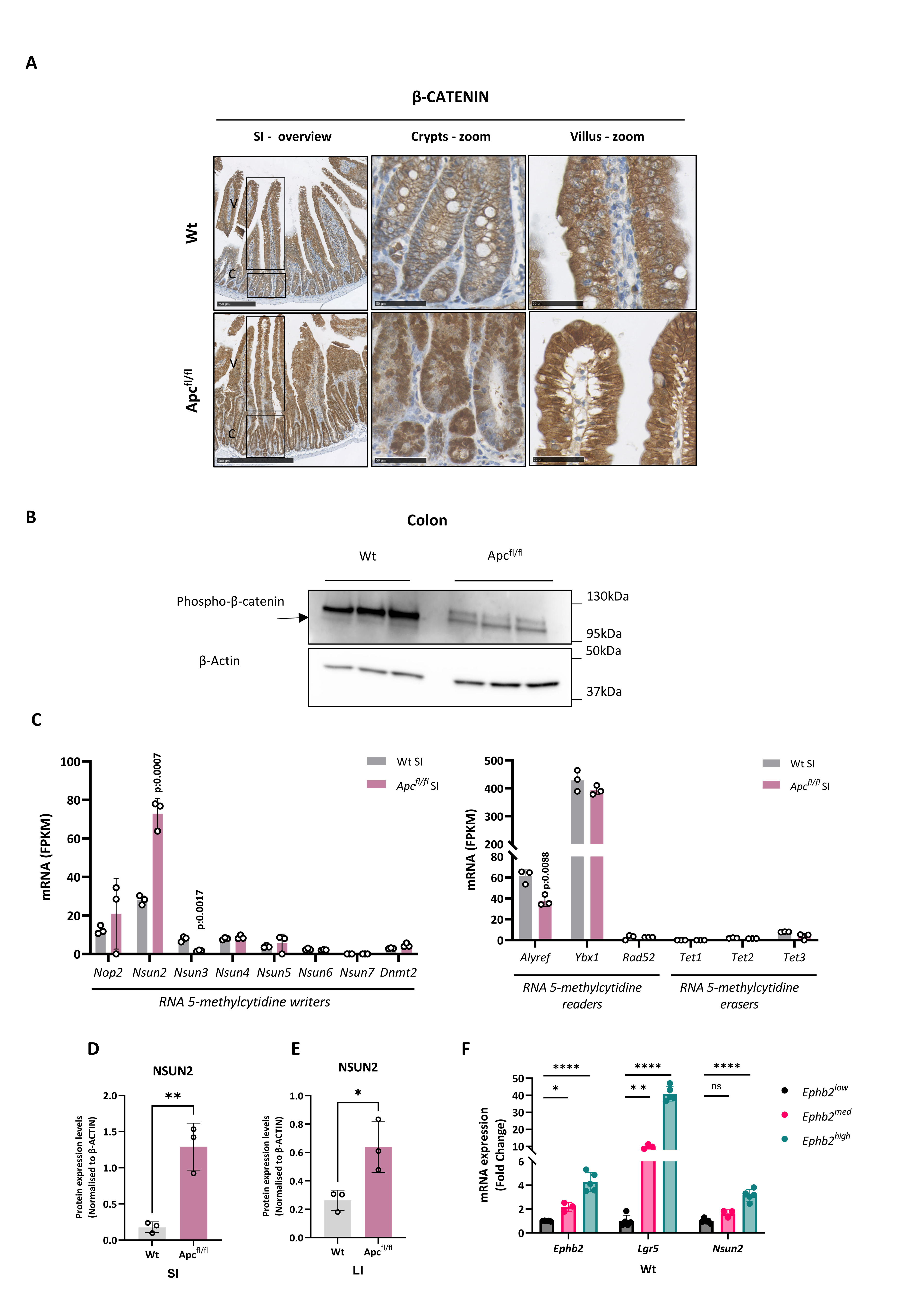

Supplement: Supplementary file 2 — Supplementary Figure S1 [file 41419_2026_8560_MOESM2_ESM.tif]

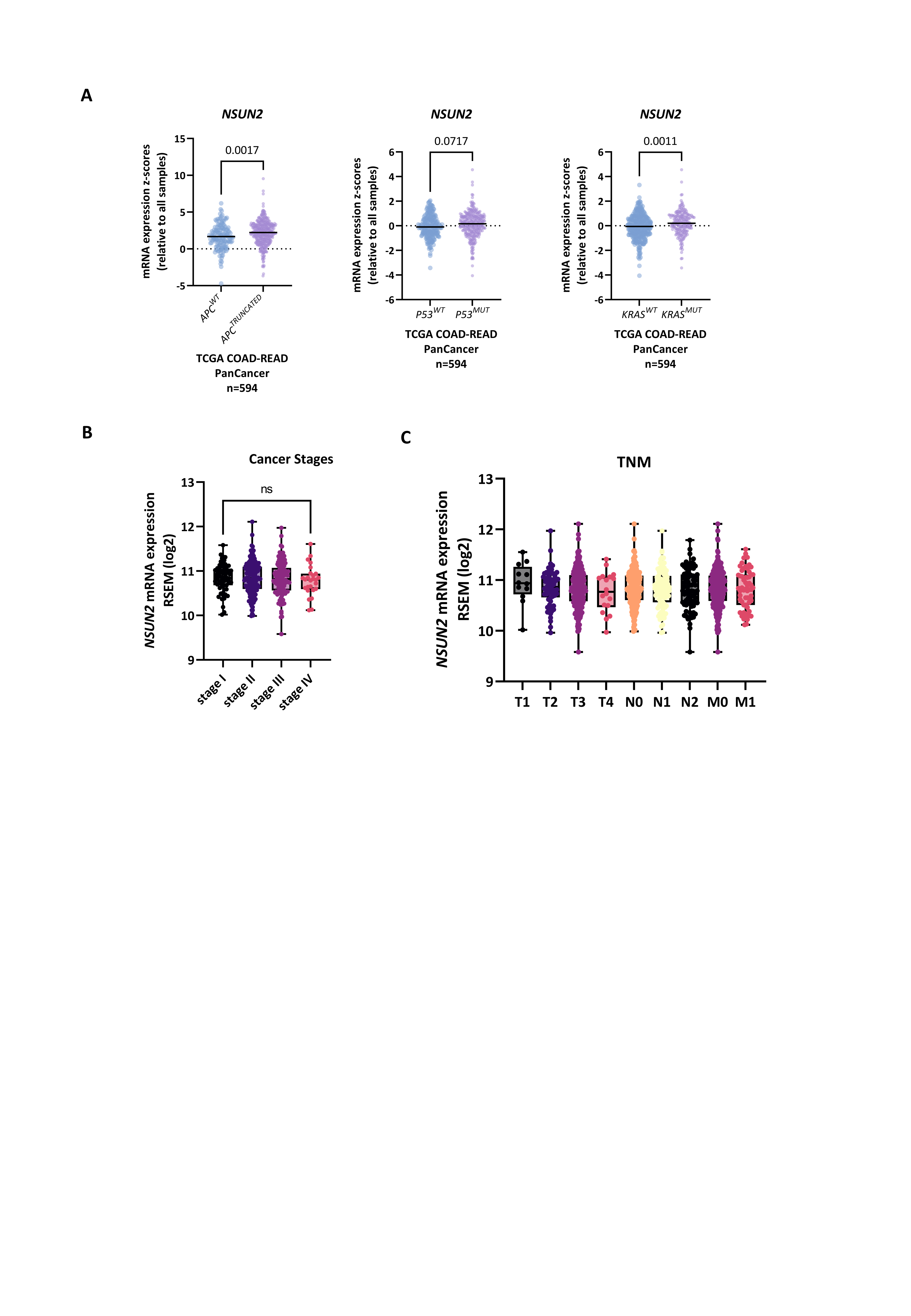

Supplement: Supplementary file 3 — Supplementary Figure S2 [file 41419_2026_8560_MOESM3_ESM.tif]

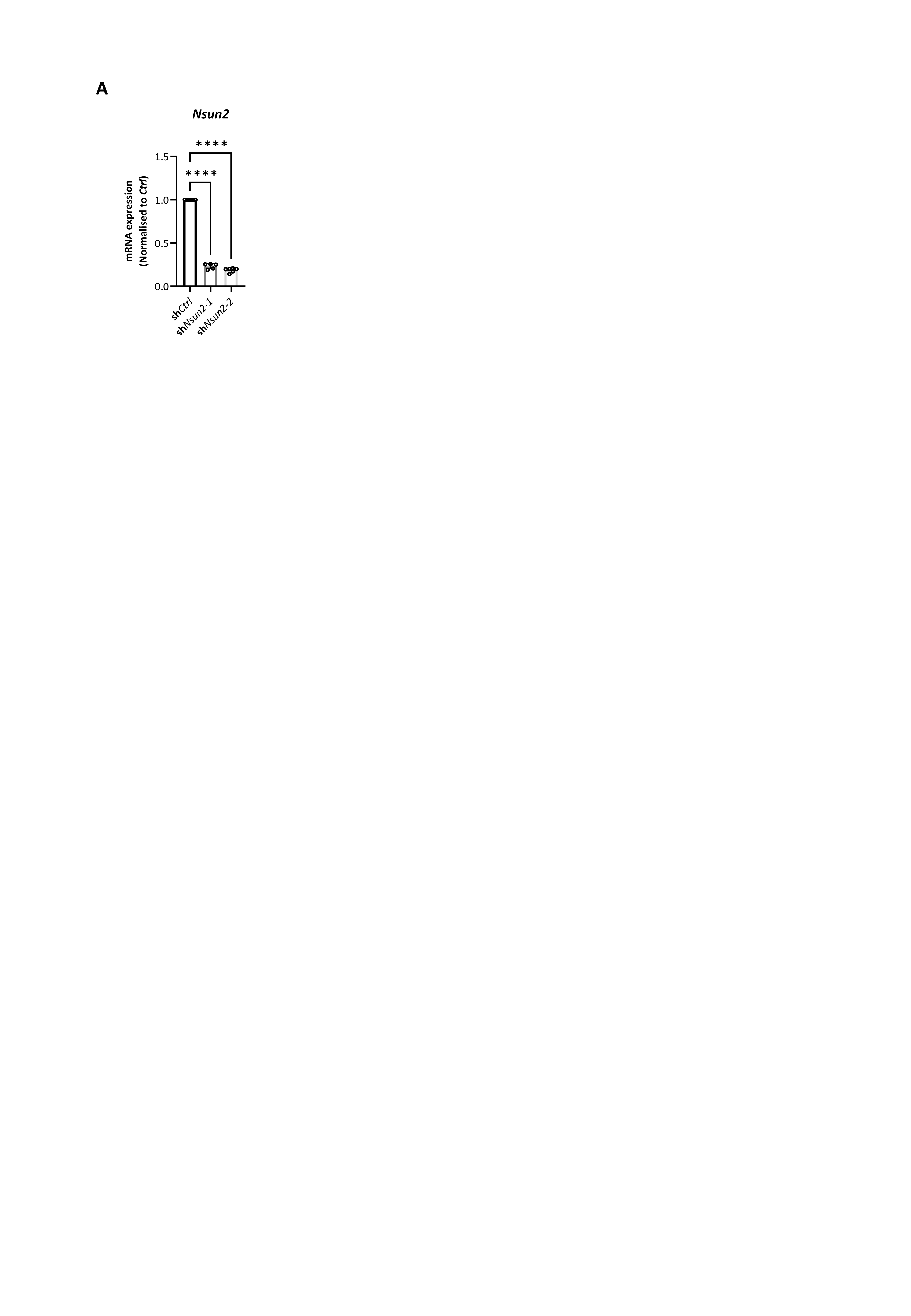

Supplement: Supplementary file 4 — Supplementary Figure S3 [file 41419_2026_8560_MOESM4_ESM.tif]

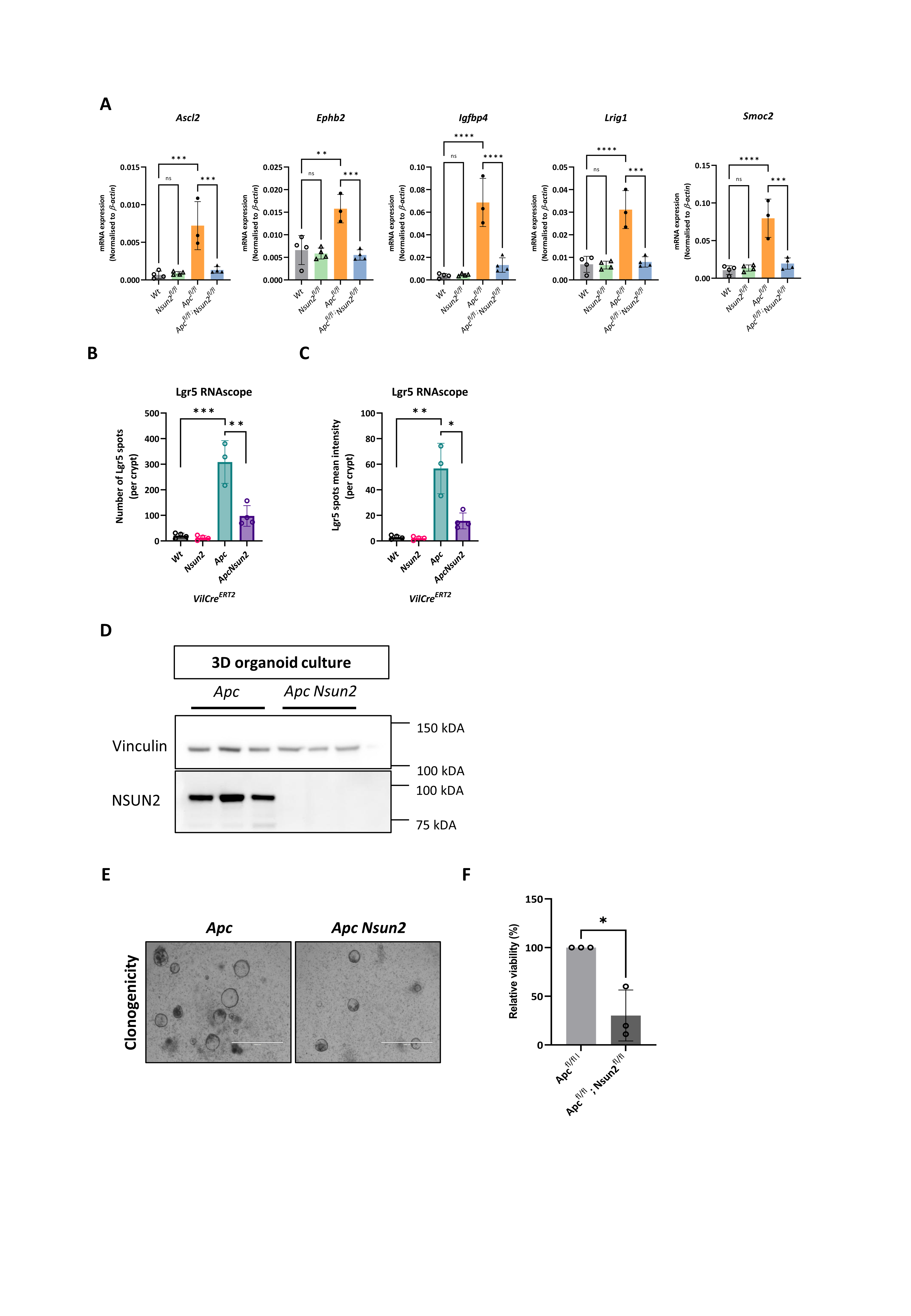

Supplement: Supplementary file 5 — Supplementary Figure S4 [file 41419_2026_8560_MOESM5_ESM.tif]

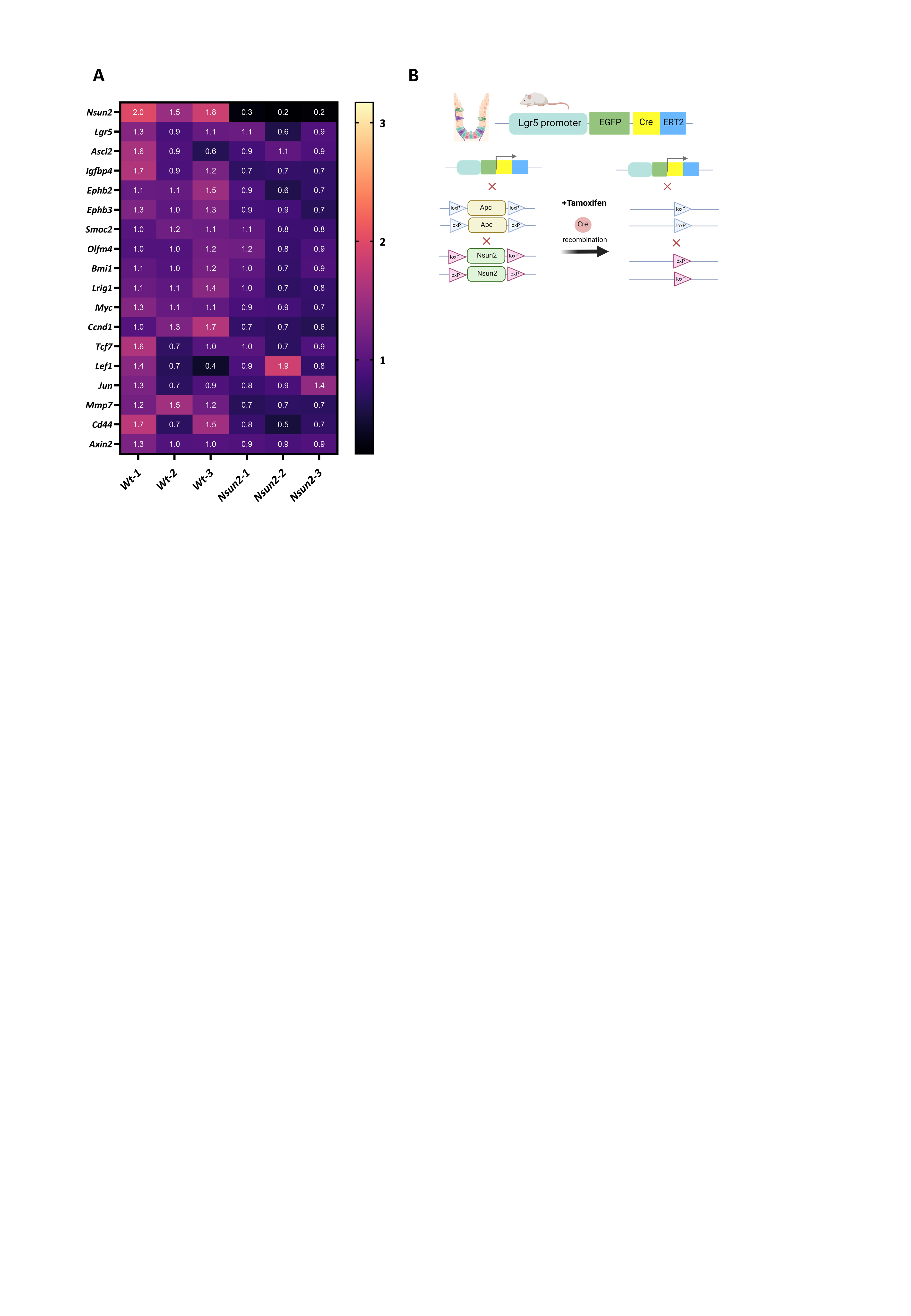

Supplement: Supplementary file 6 — Supplementary Figure S5 [file 41419_2026_8560_MOESM6_ESM.tif]

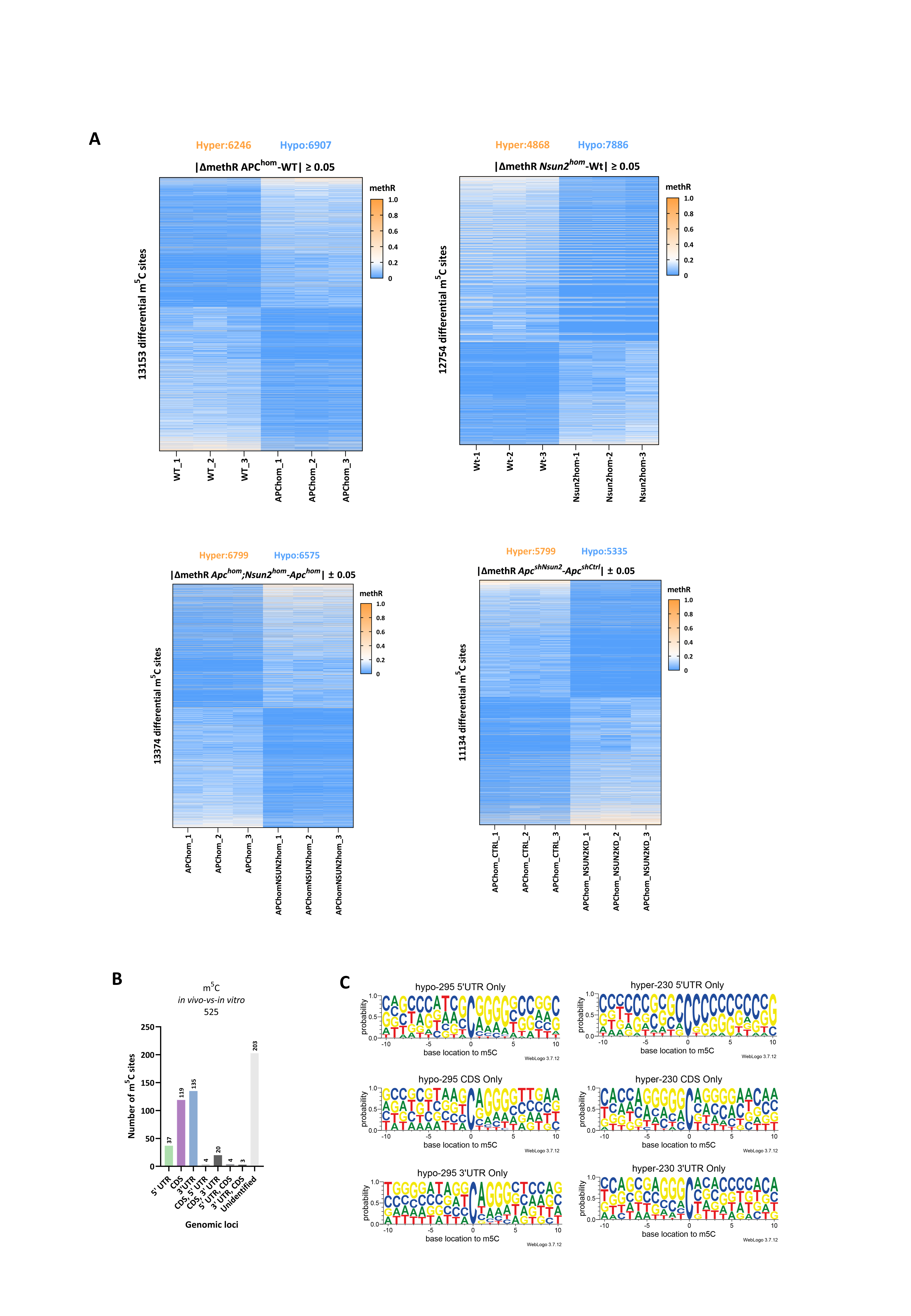

Supplement: Supplementary file 7 — Supplementary Figure S6 [file 41419_2026_8560_MOESM7_ESM.tif]

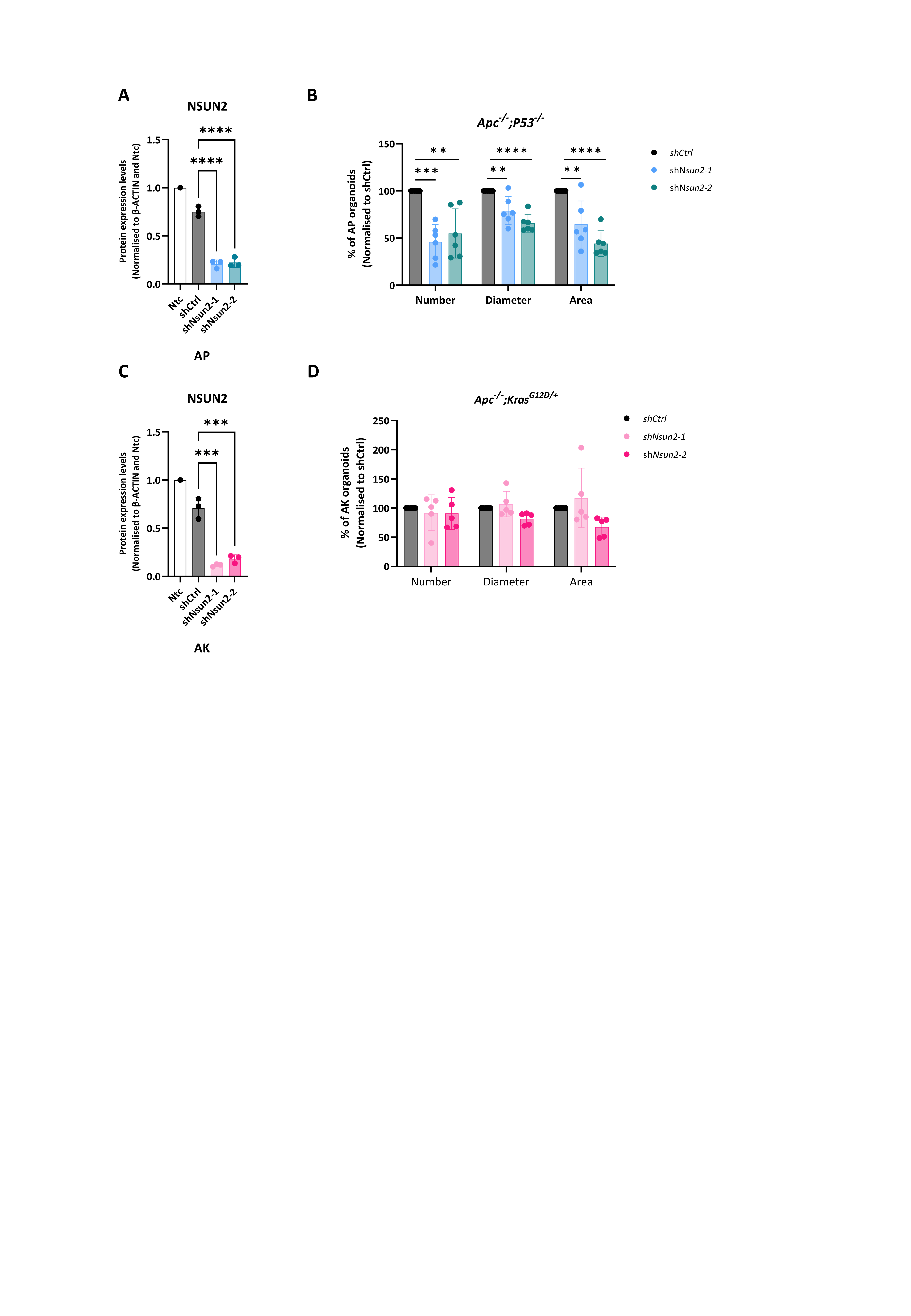

Supplement: Supplementary file 8 — Supplementary Figure S7 [file 41419_2026_8560_MOESM8_ESM.tif]

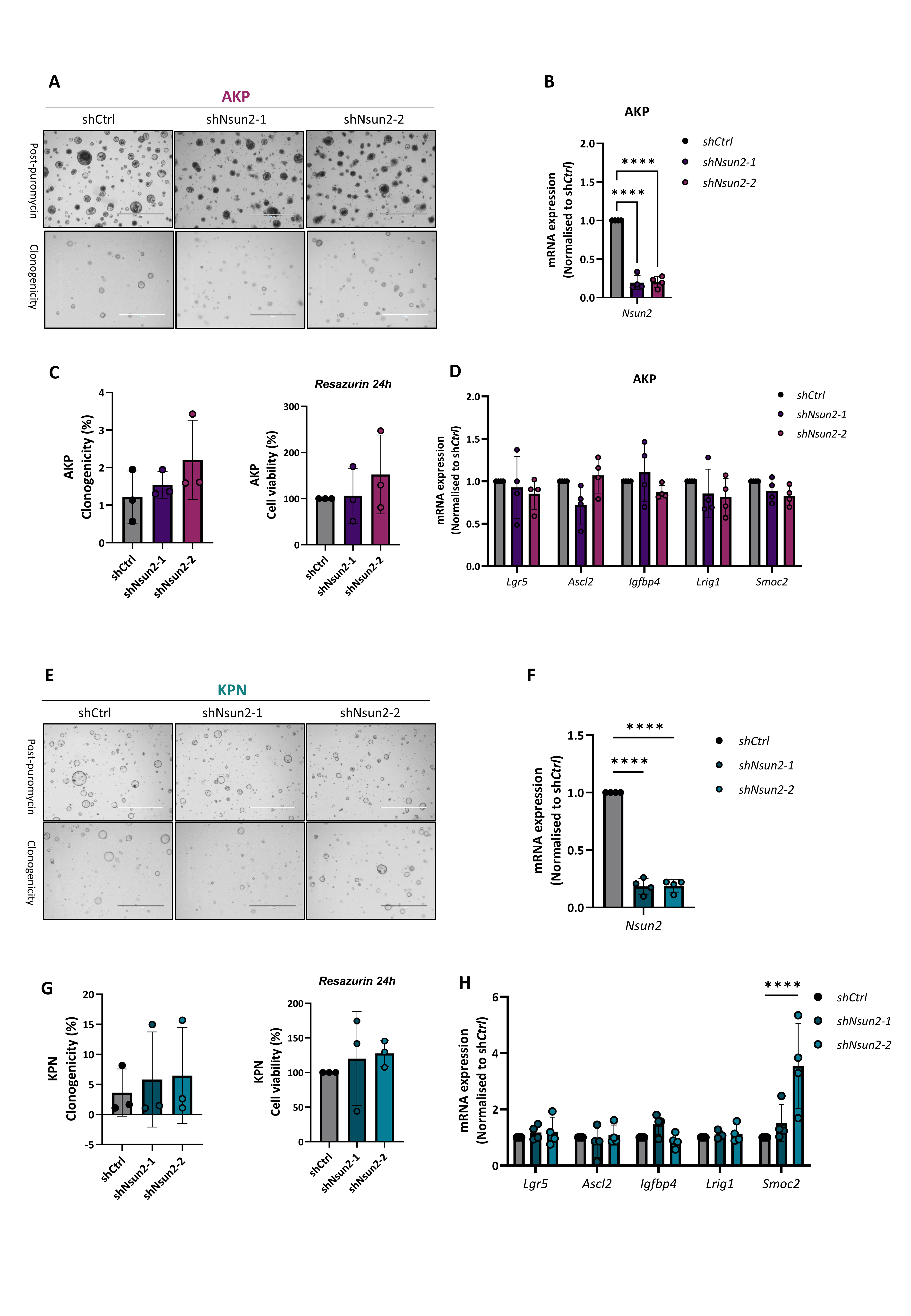

Supplement: Supplementary file 9 — Supplementary Figure S8 [file 41419_2026_8560_MOESM9_ESM.tif]

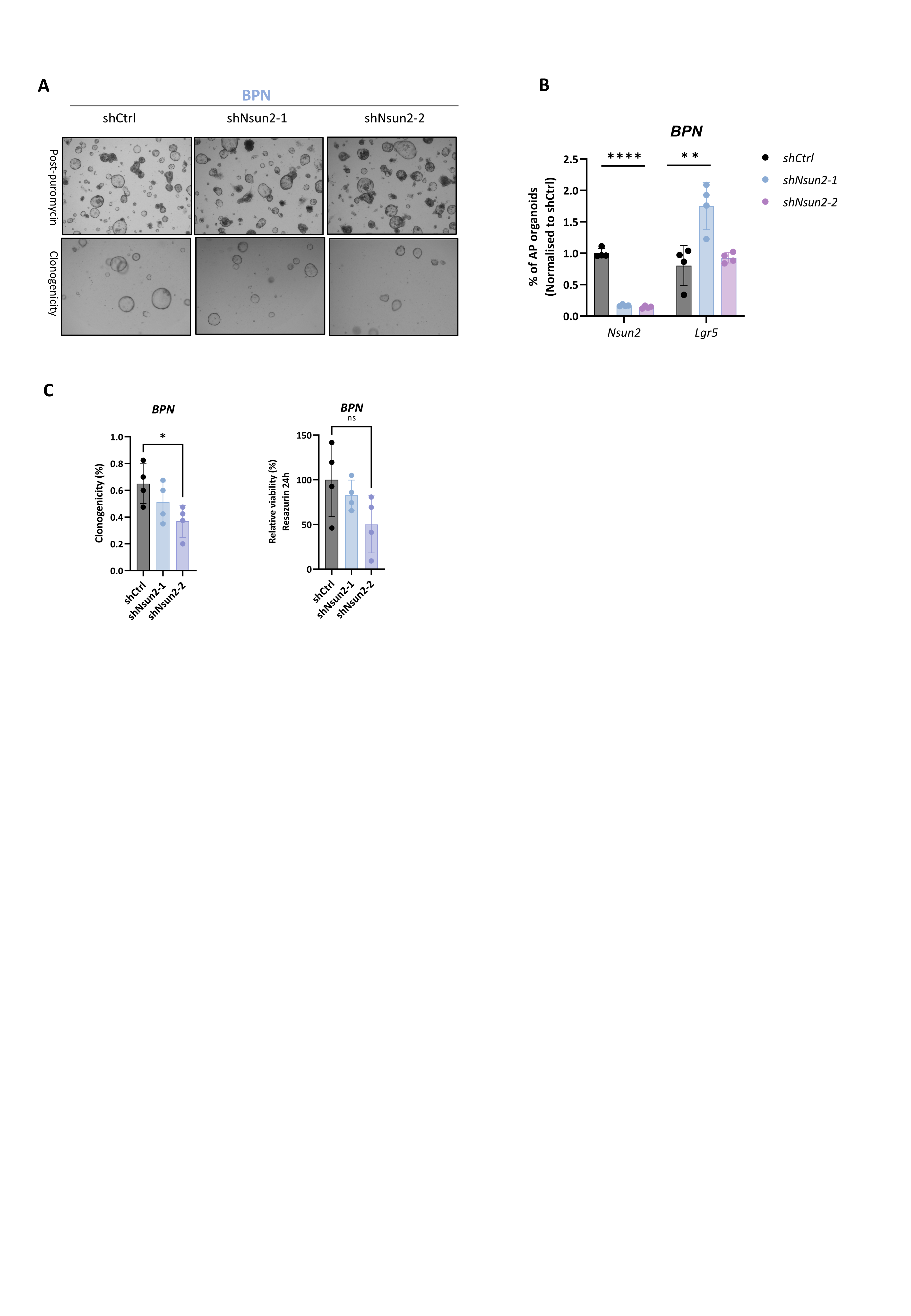

Supplement: Supplementary file 10 — Supplementary Figure S9 [file 41419_2026_8560_MOESM10_ESM.tif]
