# Supplementary material for: NSUN2 mediates intestinal stem cell expansion and colorectal tumour initiation via MAPK/ERK signalling
Source: Cell Death Dis. 2026 Mar 19;17(1):322. doi: 10.1038/s41419-026-08560-0 (PMC13039175; doi:10.1038/s41419-026-08560-0)
Supplement: Supplementary file 12 — Raw Western blot images [file 41419_2026_8560_MOESM12_ESM.pptx]

## Slide 1
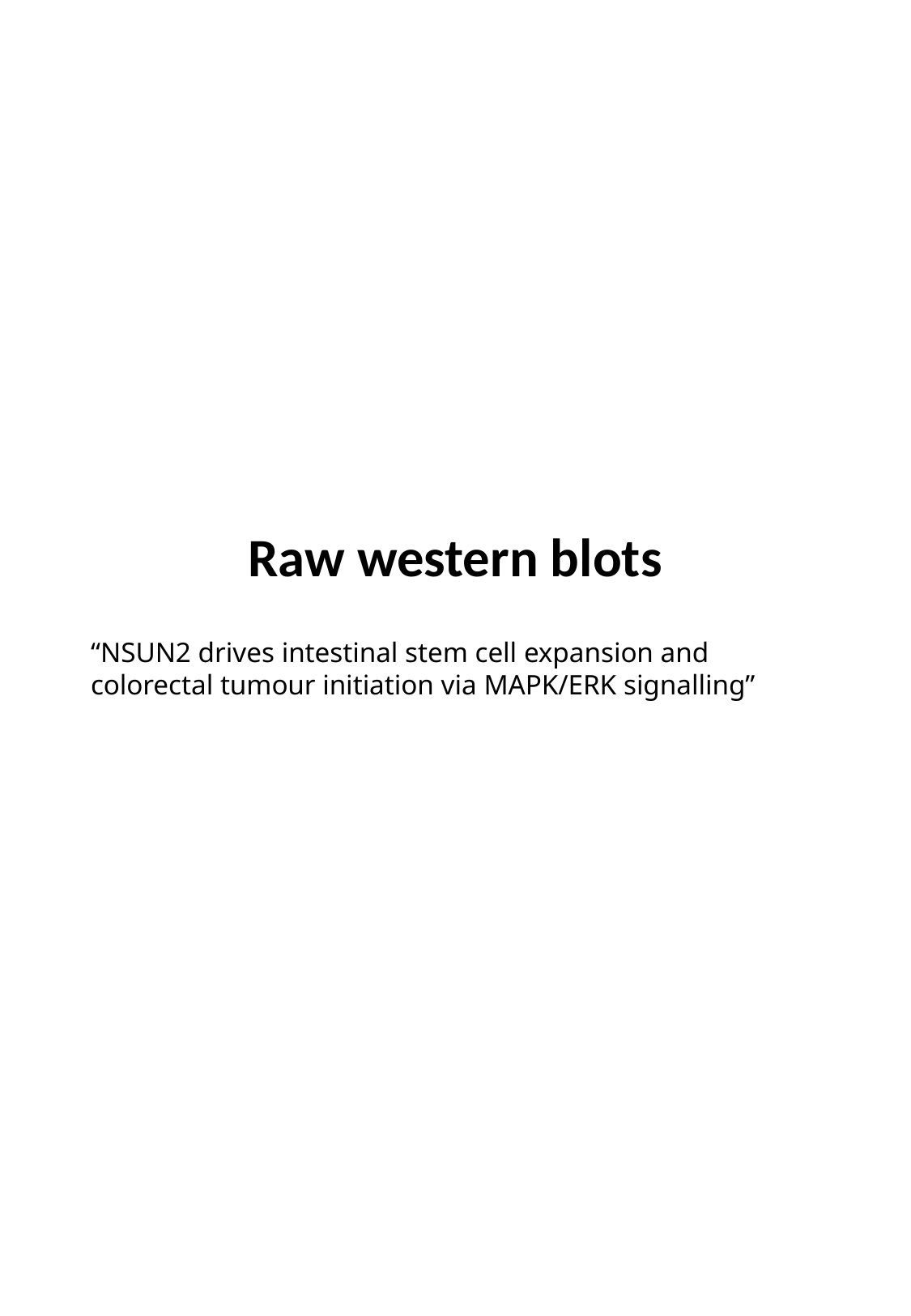

Raw western blots
“NSUN2 drives intestinal stem cell expansion and colorectal tumour initiation via MAPK/ERK signalling”

## Slide 2
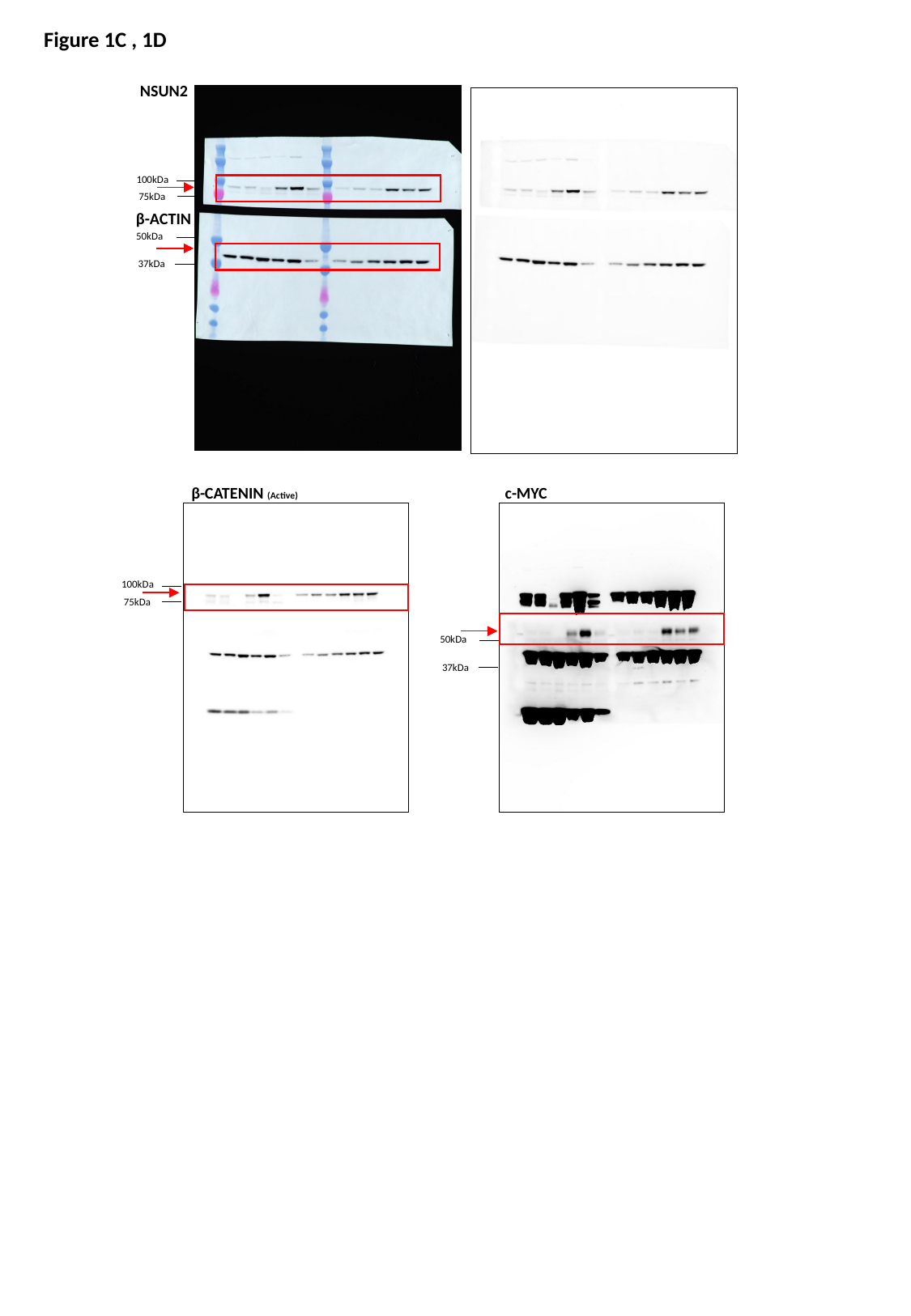

Figure 1C , 1D
NSUN2
100kDa
75kDa
β-ACTIN
50kDa
37kDa
c-MYC
β-CATENIN (Active)
100kDa
75kDa
50kDa
37kDa

## Slide 3
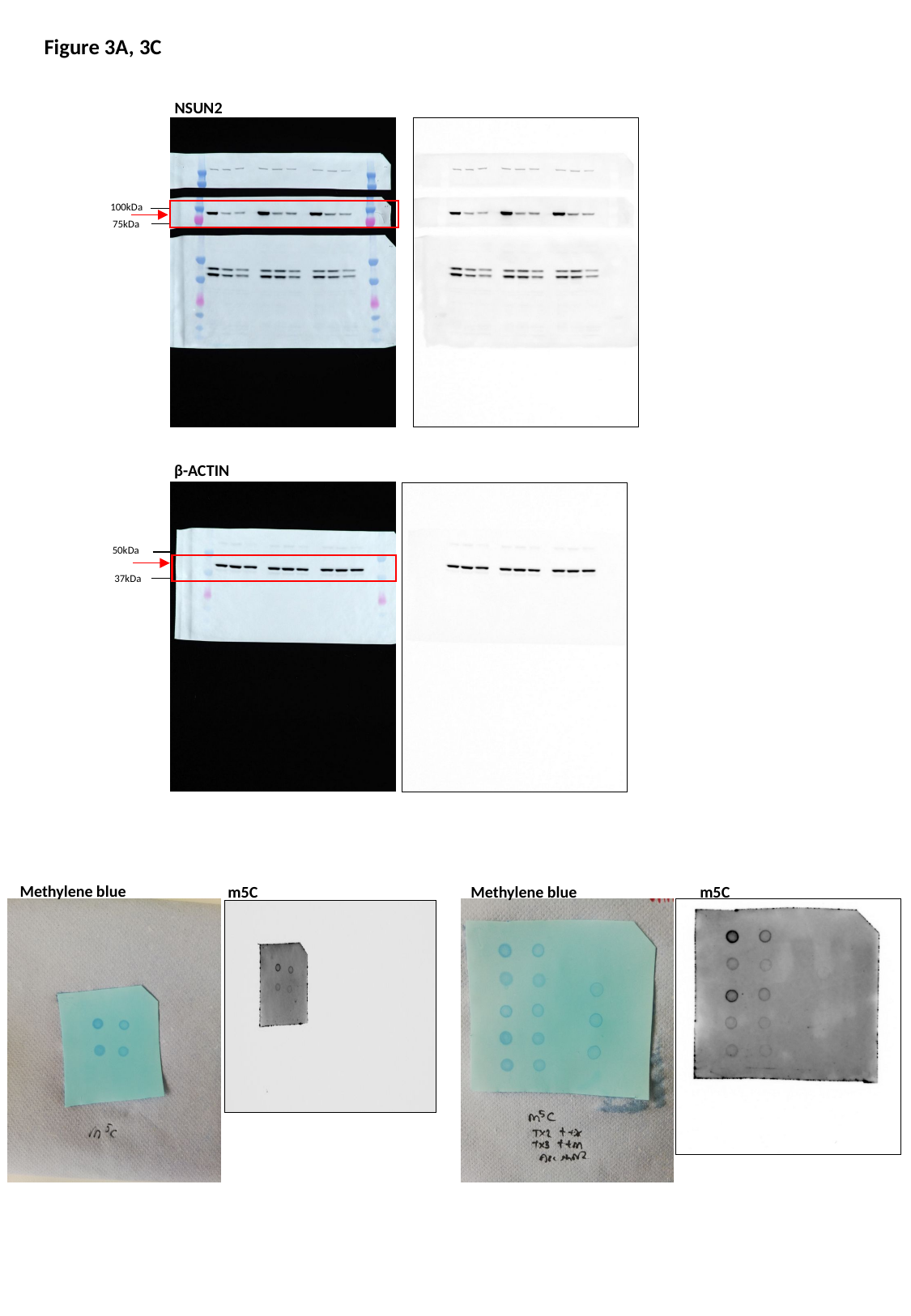

Figure 3A, 3C
NSUN2
100kDa
75kDa
β-ACTIN
50kDa
37kDa
Methylene blue
m5C
Methylene blue
m5C

## Slide 4
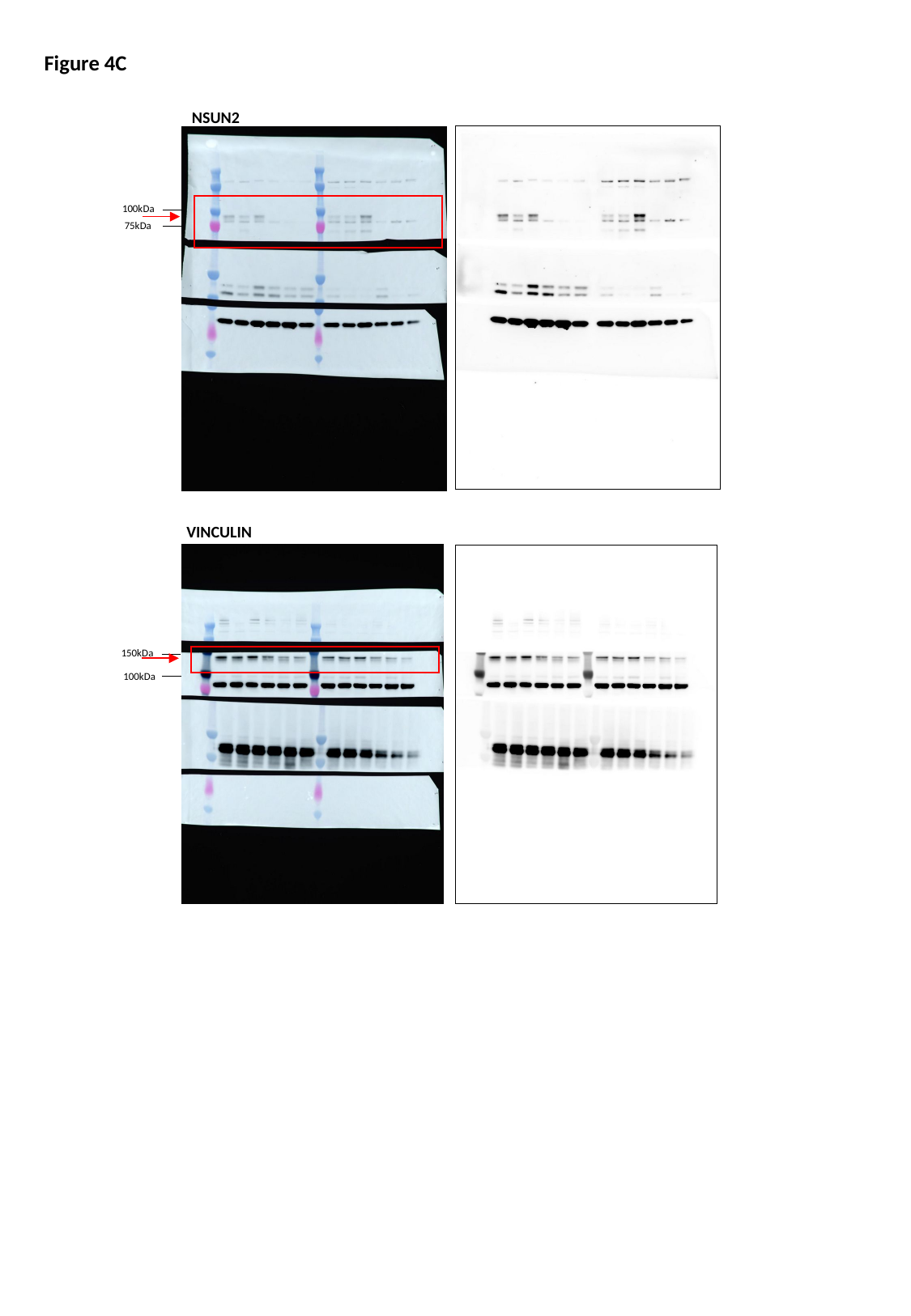

Figure 4C
NSUN2
100kDa
75kDa
VINCULIN
150kDa
100kDa

## Slide 5
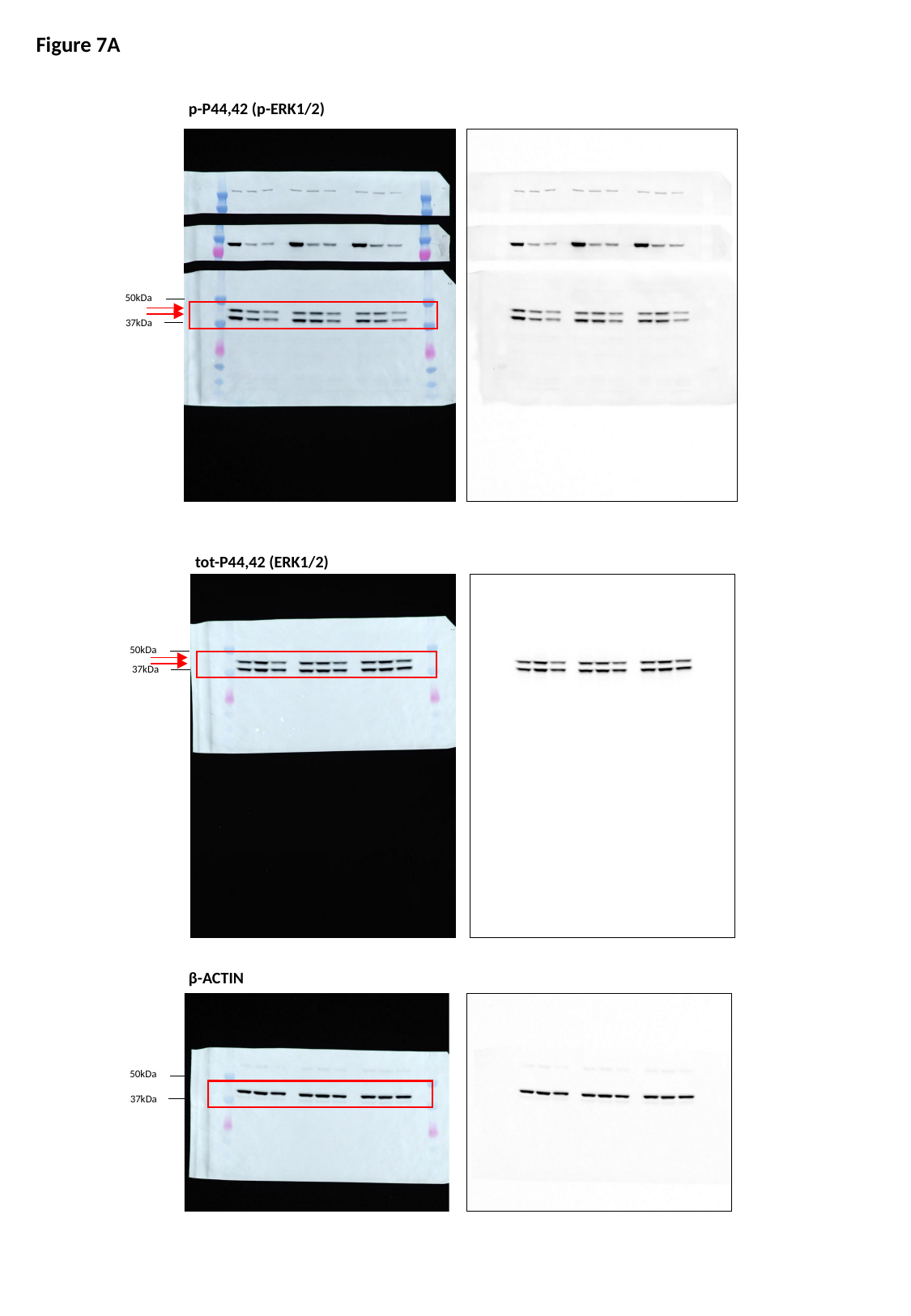

Figure 7A
p-P44,42 (p-ERK1/2)
50kDa
37kDa
tot-P44,42 (ERK1/2)
50kDa
37kDa
β-ACTIN
50kDa
37kDa

## Slide 6
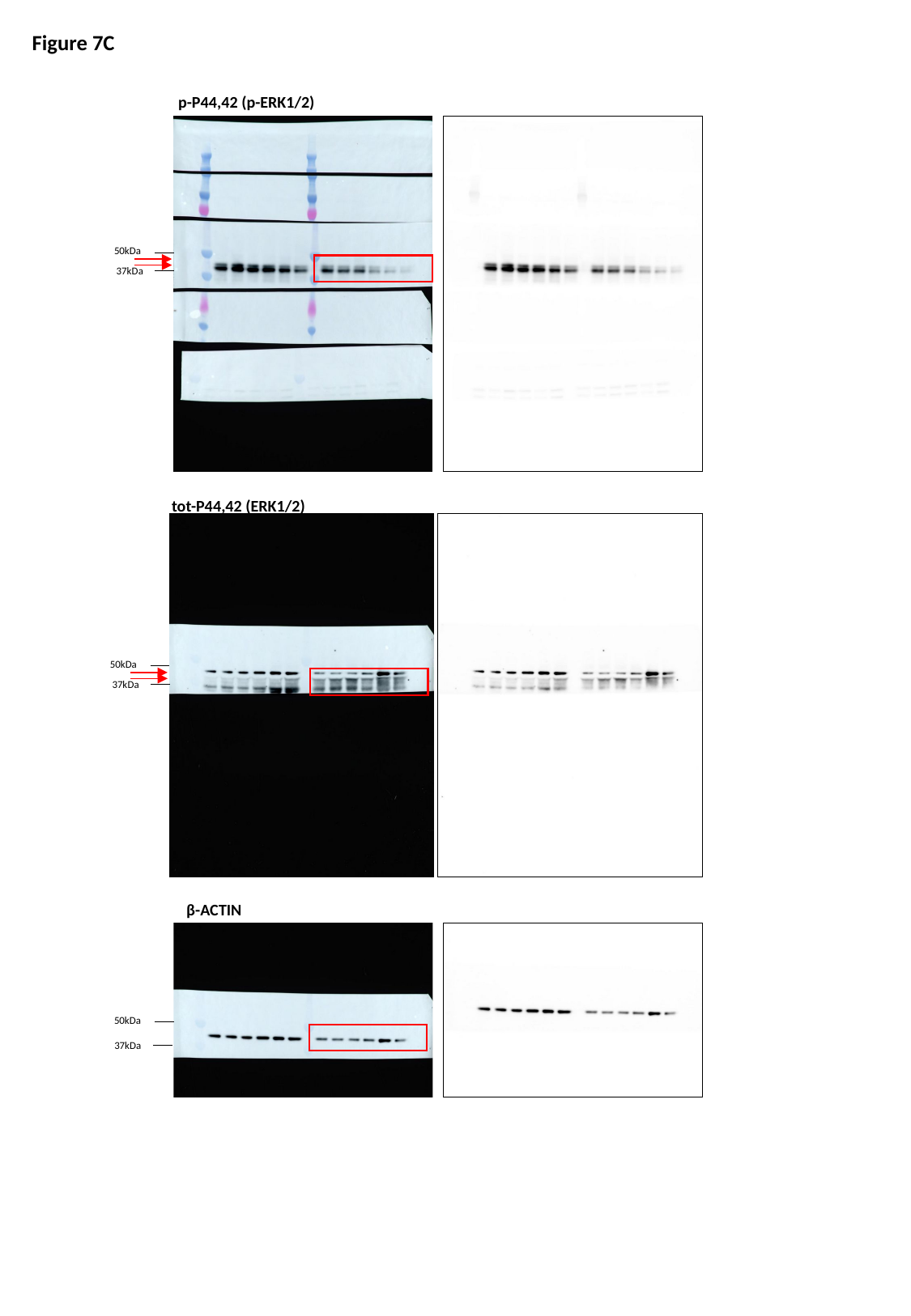

Figure 7C
p-P44,42 (p-ERK1/2)
50kDa
37kDa
tot-P44,42 (ERK1/2)
50kDa
37kDa
β-ACTIN
50kDa
37kDa

## Slide 7
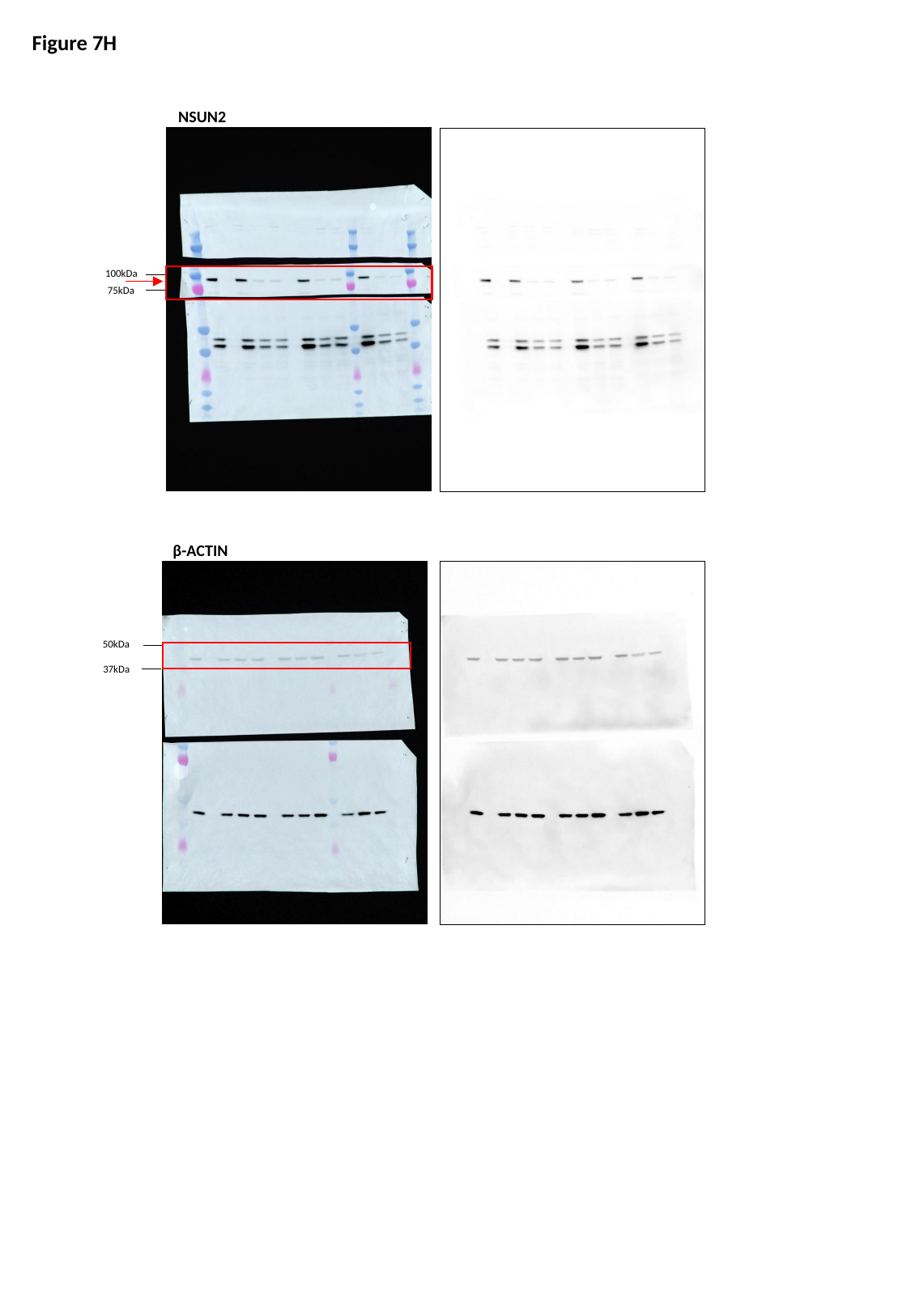

Figure 7H
NSUN2
100kDa
75kDa
β-ACTIN
β-ACTIN
50kDa
37kDa

## Slide 8
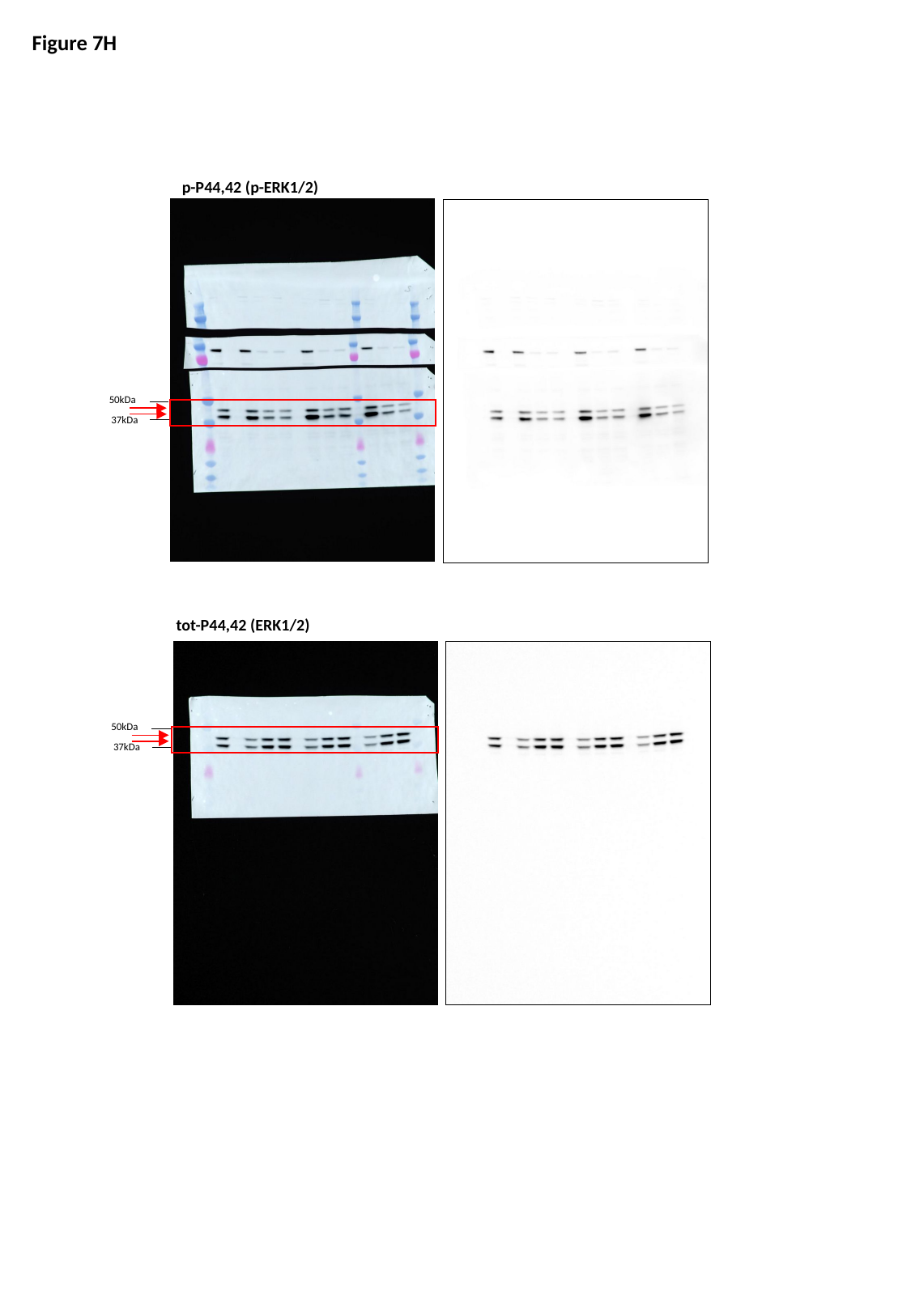

Figure 7H
p-P44,42 (p-ERK1/2)
50kDa
37kDa
tot-P44,42 (ERK1/2)
50kDa
37kDa

## Slide 9
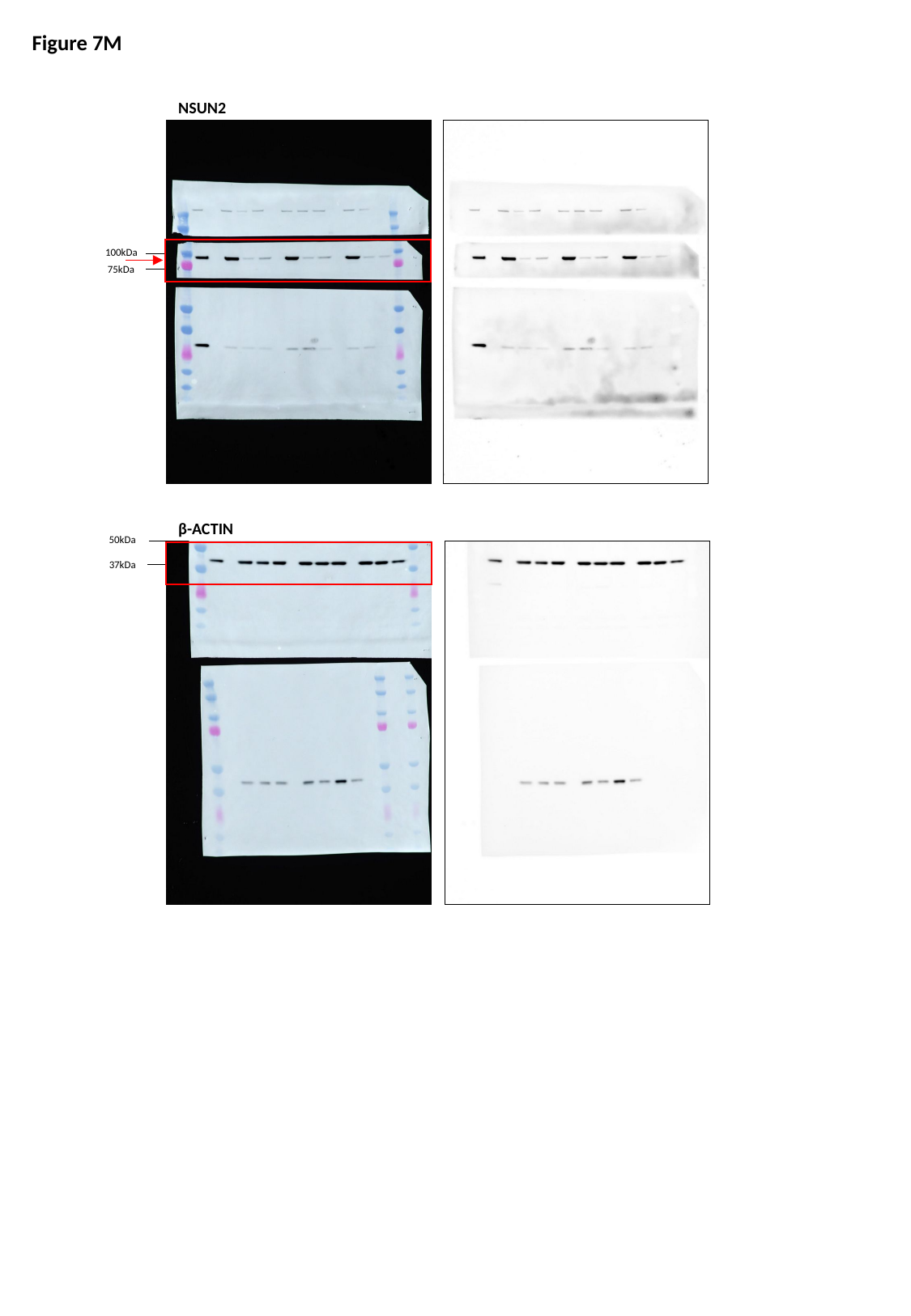

Figure 7M
NSUN2
100kDa
75kDa
β-ACTIN
50kDa
37kDa

## Slide 10
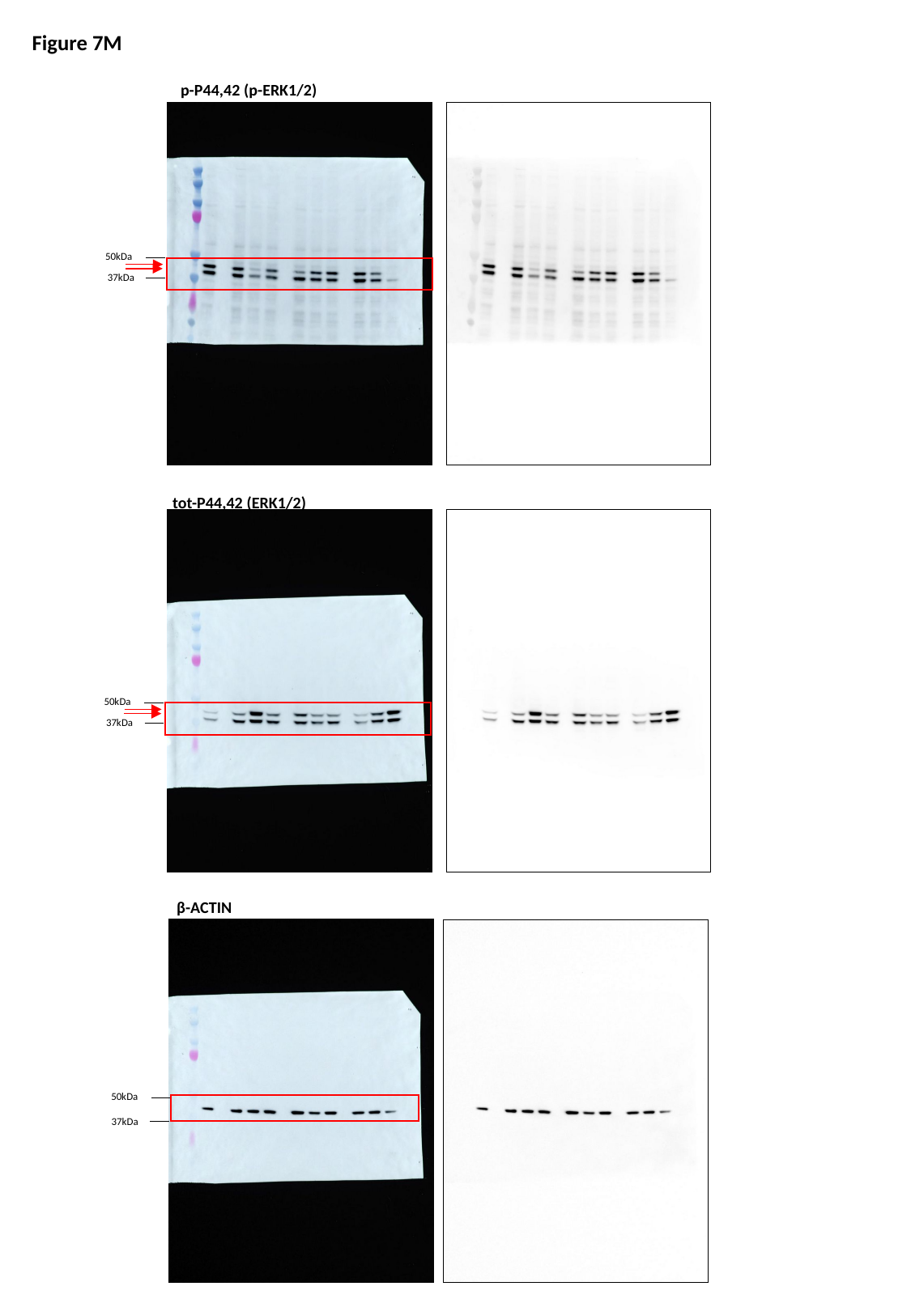

Figure 7M
p-P44,42 (p-ERK1/2)
50kDa
37kDa
tot-P44,42 (ERK1/2)
50kDa
37kDa
β-ACTIN
50kDa
37kDa

## Slide 11
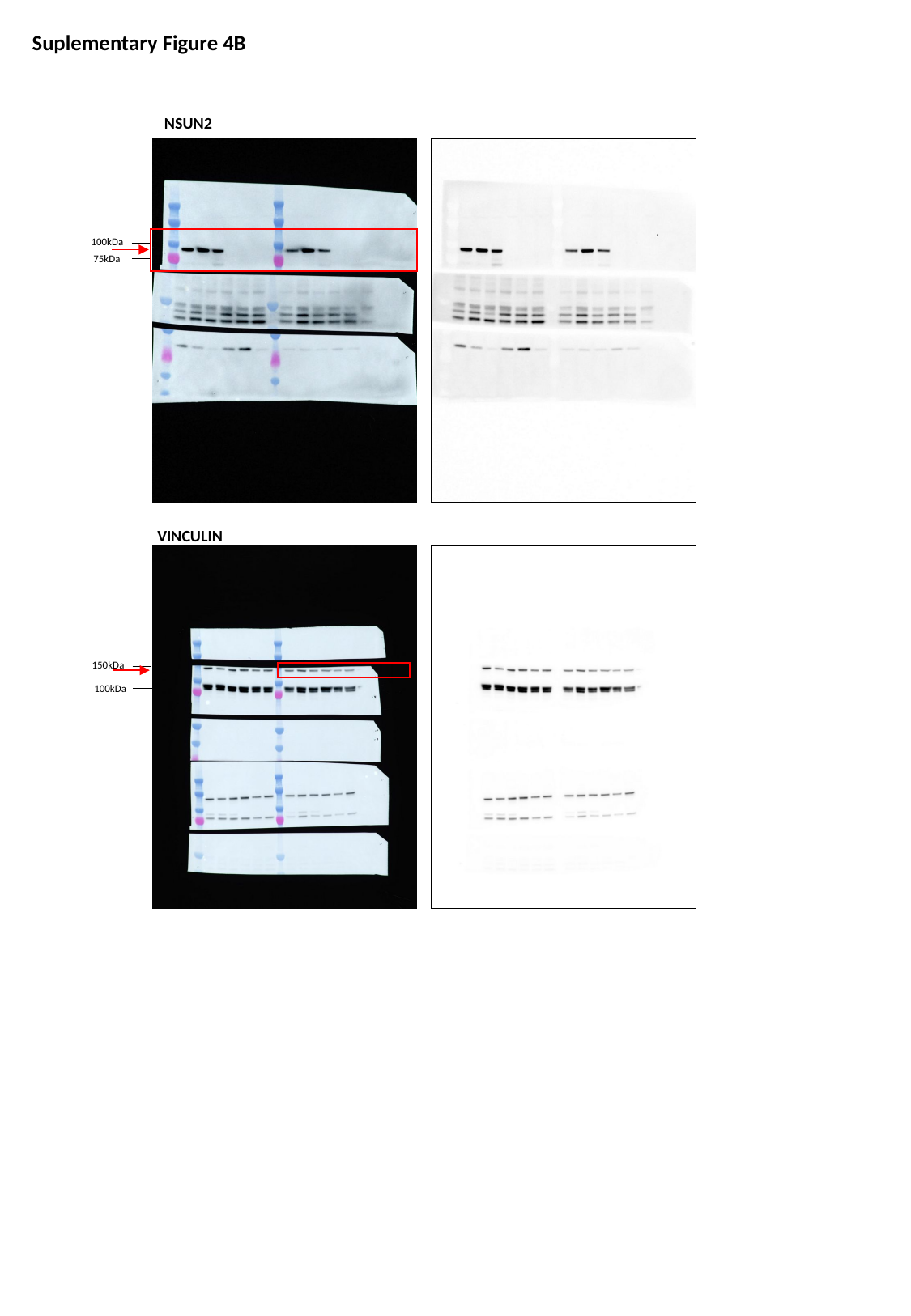

Suplementary Figure 4B
NSUN2
100kDa
75kDa
VINCULIN
150kDa
100kDa
